# Is there a relationship between the severity of obstructive sleep apnea syndrome and the systemic immune inflammation index?

**DOI:** 10.1007/s00405-024-08729-3

**Published:** 2024-05-18

**Authors:** Meltem Karacan Gölen, Şaziye Melike Işık, Veysel Arıkan

**Affiliations:** 1 Department of Neurology, Baskent University Hospital Konya, Selcuklu, Konya Turkey 9042080; 2 Department of Neurology, Konya Numune State Hospital, Selcuklu, Konya Turkey 9042060; 3 Department of Pulmoner Disease, Konya Numune State Hospital, Selcuklu, Konya Turkey 9042060

**Keywords:** Obstructive sleep apnea syndrome (OSAS), Systemic immune inflammation index (SII), NLR, PLR

## Abstract

**Aim:**

Vascular dysfunction, oxidative stress and systemic inflammation are considered responsible for the pathophysiology of Obstructive sleep apnea syndrome (OSAS). It is thought that desaturation due to apnea–hypopnea attacks in OSAS patients activates inflammatory pathways. In this study, we aimed to reveal the relationship between inflammation parameters Systemic immune inflammation index (SII), neutrophil-to-lymphocyte ratio (NLR) and platelet-to-lymphocyte ratios (PLR) severity of OSAS in patients who underwent polysomnography in our hospital’s sleep laboratory.

**Methods:**

We grouped our 171 patients who were followed up in our sleep laboratory with the diagnosis of OSAS according to their AHI values. We evaluated the correlation of SII, NLR, and PLR values obtained from the complete blood tests of our patients with OSAS diagnosis and OSAS severity.

**Results:**

The mean NLR, PLR and SII values of patients with OSAS were statistically significantly higher than those without OSAS (p < 0.05). A positive correlation of 18% was found between the presence of OSAS and the SII value (p = 0.016). No statistically significant difference was found when comparing OSAS severity and NLR, PLR and SII values (p > 0.05).

**Conclusion:**

We observed that SII, NLR and PLR parameters, which are rapidly assessable systemic inflammation markers of this process, were independently associated in patients diagnosed with OSAS and that there was no change in SII, NLR, and PLR parameters with OSAS severity.

## Introduction

Obstructive sleep apnea syndrome (OSAS) is a chronic inflammatory syndrome characterized by recurrent complete (apnea) or partial (hypopnea) upper airway obstruction during sleep, resulting in decreased blood oxygen saturation during sleep and increased daytime sleepiness [1].

Vascular dysfunction, oxidative stress and systemic inflammation are considered responsible for the pathophysiology of OSAS. It is thought that desaturation due to apnea–hypopnea attacks in OSAS patients activates inflammatory pathways. Accordingly, studies have shown the presence of systemic inflammation in OSAS patients by markers such as C-reactive protein (CRP), leptin, interleukin-6 (IL-6), interleukin-8 (IL-8), tumour necrosis factor-alpha (TNF-a), and vascular endothelial growth factor (VEGF) [2]. In addition, the deoxygenation occurring during the cycle affects the tissues, causing endothelial dysfunction and increasing the systemic inflammatory response. This causes atherosclerotic changes by causing the interaction of platelets, lymphocytes and neutrophils with endothelial cells. This chronic inflammation is the most significant risk factor for atherosclerosis and cardiovascular diseases. Due to this pathophysiological process, it is thought that the increased systemic inflammatory response in patients with OSAS increases the risk of coexistence of primarily cardiovascular diseases, pulmonary diseases and neuropsychological diseases [3].

Systemic immune inflammation index (SII), first proposed by Wu et al., is an infectious marker measured by complete blood count. In addition to SII, neutrophil-to-lymphocyte ratio (NLR) and platelet-to-lymphocyte ratio (PLR) have recently been used as markers associated with inflammation in many diseases. They are widely used because they are low-cost, easily accessible, and derived from complete blood count parameters [4–6].

In this study, we aimed to reveal the relationship between inflammation parameters SII, NLR and PLR ratios and OSAS and the severity of OSAS in patients who underwent polysomnography in our hospital’s sleep laboratory.

## Methods

Patients aged 18–70 who underwent polysomnography in our hospital's sleep laboratory between April 2022 and December 2023 and had one or more of the symptoms of snoring during sleep, excessive daytime sleepiness, and witnessed apnea were included.

Patients with systemic inflammatory disease, malignancy and active infection were excluded from the study. The patients’ habits, chronic diseases, demographic data and body mass index (BMI) were recorded. Epworth sleepiness scale was applied to the patients before PSG.

The Philips Respironics Alice 5 Diagnostic Sleep System monitored sleep and physiological variables. Electroencephalography with 10 channels (C3, C4, O1, O2, Fp1, Fp2, F3, F4, P3, P4), submental electromyography (EMG), right and left eye electrooculography, electrocardiography, oronasal airflow (thermal sensor and nasal pressure transducer), body position, thoracic and abdominal motion meter (inductance plethysmograph), arterial blood oxygen saturation measurement with finger pulse oximetry, left and right leg movement sensors (EMG) and tracheal microphone were used. *Apnea* was defined as a decrease of more than 90% in the airflow signal measured by the thermal sensor for at least 10 s. *Hypopnea* was defined as a decrease in the nasal pressure signal of more than 30% compared to baseline and desaturation of more than 3% compared to baseline for at least 10 s, or resulting in arousal. Apnea–hypopnea index (AHI) 5–14.9 was accepted as mild OSAS, AHI: 15–29.9 as moderate OSAS, AHI ≥ 30 as severe OSAS, and AHI < 5 and snoring as simple snoring.

Peripheral blood evaluations were performed on venous blood samples taken from the antecubital region 1 day before the PSG test. Patients’ blood was taken to investigate infection findings, and patients with active infection were not included in the study.

Laboratory markers such as White Blood Cell (WBC), neutrophil, lymphocyte, platelet and Red Cell Distribution Width (RDW) values were recorded. NLR = neutrophil-to-lymphocyte ratio, PLR = platelet-to-lymphocyte, Systemic immune-inflammation index (SII) was calculated according to the Neutrophil count x Platelet count / Lymphocyte count ratio.

Karatay University Local Ethics Committee approval was received. (2024/028).

### Statistical analysis

The data from the study were analyzed on a computer using the SPSS (Statistical Package for Social Sciences) 27.0 package program. The t-test was used to compare numerical data in two independent groups, and the ANOVA test was used to compare numerical data in more than two independent groups. The Levene test evaluated the homogeneity of variances. When there was a significant difference between groups in the ANOVA test, post-hoc comparisons of pairs were made using the Scheffe test if the variances were homogeneous and the Tamhane T2 test if the variances were not homogeneous. The relationship between a numerical variable and a qualitative variable was evaluated using Eta correlation coefficient. p < 0.05 was considered significant.

## Results

64.3% of the participants were male, and 67.8% were obese. 41.7% of women were in menopause. 22.2% of the participants had hypertension, 17.0% had diabetes, 5.9% had hyperlipidemia, 10.5% had coronary artery disease, 21.6% had COPD or asthma, 6.4% had CVA, and 2.3% had epilepsy. In 42.1% of the participants, increased daytime sleepiness was found, and normal EPW was found in 31%. 76% of the participants had OSAS, and 39.2% were considered to have severe OSAS (Table 1).

**Table 1:** Demographics of patients with OSAS

Characteristics	Group	N	%
Gender	Man	110	64.3
	Woman	61	35.7
Obesity status	Normal	6	3.5
	Overweight	49	28.7
	Obese	116	67.8
Menopause	Yes/No	25/36	41.0/59
Hypertension	Yes/No	38/133	22.2/77.8
Diabetes Mellitus	Yes/No	29/142	17.0/83.0
Hyperlipidemia	Yes/No	10/161	5.8/94.2
Coronary Artery Disease	Yes/No	18/153	10.5/89.5
COPD/Asthma	Yes/No	37/134	21.6/78.4
Cerebrovascular Disease	Yes/No	11/160	6.4/93.6
Epilepsy	Yes/No	4/167	2.3/97.7
Daytime Sleepiness (ESS)	Normal Increased	99 72	57.9 42.1
OSAS	Yes/No	130/41	76.0/24
OSAS Severity	Simple Snoring	41	24.0
	Mild OSAS	41	24.0
	Moderate OSAS	22	12.9
	Severe OSAS	67	39.2

*OSAS* obstructive sleep apnea syndrome, *AHI* Apnea–Hypopnea Index, *ESS* Epworth Sleepiness Scale

The average age of the participants was 44.55 ± 12.42 kg/m^2^, and the average BMI was 33.35 ± 6.17 kg/m^2^.

The mean of Epworth scores was 9.63 ± 6.47, the mean REM latency was 194.59 ± 84.34, the mean AHI was 24.57 ± 22.56, the mean REM AHI was 23.78 ± 26.99, the mean Non-REM AHI was 24.28 ± 22.52 (Table 2).

**Table 2:** Evaluation of sleep recording data

	Mean ± SD	Min–Max
Age	44.55 ± 12.42	18.00–78.00
Height	1.69 ± 0.09	1.47–1.95
Weight	95.69 ± 18.93	54.00–153.00
BMI (kg/m^2^)	33.35 ± 6.17	18.25–58.59
ESS	9.63 ± 6.47	0.00–24.00
Total sleep time	380.82 ± 61.71	118.00–549.50
Sleep efficiency (%)	82.08 ± 11.27	23.80–98.70
Sleep latency	31.00 ± 31.43	0.00–184.50
REM latency	194.59 ± 84.34	0.00–424.50
N-REM 1 (%)	2.61 ± 4.10	0.00–22.50
N-REM 2 (%)	82.40 ± 61.10	31.60–342.00
N-REM 33 (%)	39.13 ± 33.59	0.00–255.00
REM (%)	15.46 ± 15.30	0.00–107.00
AHI	24.57 ± 22.56	0.00–99.6
REM AHI	23.78 ± 26.99	0.00–106.00
Mean SPO_2_ (%)	90.94 ± 5.39	40.00–98.00
Min SPO_2_ (%)	75.79 ± 12.95	27.00–93.00

*OSAS* obstructive sleep apnea syndrome, *AHI* Apnea–Hypopnea Index, *BMI* body mass index, *ESS* Epworth Sleepiness Scale, *Min.SpO*_*2*_ minimum oxygen saturation

As shown in Table 3, the participants’ mean PLT was 271.13 ± 66.61, mean HGB was 14.57 ± 1.56, mean RDW was 13.42 ± 1.36, mean NEU was 5.12 ± 2.06, mean LYM was 2.65 ± 0.83, mean NLR 2.21 ± 1.90, mean PLR was 111.45 ± 44.45, and mean SII was 575.87 ± 369.99.

**Table 3:** Laboratory variables

Platelet, × 10^3^/μL	271.130 ± 66.610	210.180–488.000
Hemoglobin g/dL	14.57 ± 1.56	10.80–18.10
RDW%	13.42 ± 1.36	11.40–21.10
Neutrophil, × 10^3^/μL	5.12 ± 2.06	1.61–16.95
Lymphocyte, × 10^3^/μL	2.65 ± 0.83	0.37–5.75
NLR	2.21 ± 1.90	0.71–21.27
PLR	111.45 ± 44.45	1.46–420.62
SII	575.87 ± 369.99	6.00–3003.24

*RDW* red cell distribution width, *NLR* neutrophil/lymphocyte ratio, *PLR* platelet/lymphocyte ratio, *SII* systemic immune inflammation index

A comparison of AHI values of the patients by OSAS severity revealed a statistically significant difference between all groups (p < 0.001). A comparison of the ages of the patients by OSAS severity showed that the average age of the patients with severe OSAS was significantly higher than the simple snoring and mild OSAS groups (p < 0.001). The mean BMI of patients with severe OSAS was significantly higher than that of patients with mild OSAS (p = 0.048). The mean SpO_2_ values of patients with severe OSAS were significantly lower than the groups with simple snoring and mild OSAS (p < 0.001) (Table 4).

**Table 4:** Comparison of OSAS severity by AHI, age, BMI and mean SpO_2_

OSAS Severity	AHI Mean ± SD (min–max)	Gender		Age (years) Mean ± SD (min–max)	BMI (kg/m^2^) Mean ± SD (min–max)	Mean SpO_2_ (%) Mean ± SD (min–max)
		Man (n)	Woman (n)			
Simple Snoring (n = 41)	2.12 ± 1.71 (0.0–7.9)	22	19	39.59 ± 12.55 (18–65)	32.26 ± 6.55 (18.25–47.23)	93.28 ± 3.00 (77.5–96.0)
Mild OSAS (n = 41)	9.29 ± 3.13 (5.0–15.5)	30	11	40.07 ± 12.49 (18–74)	31.89 ± 6.76 (19.94–51.12)	92.50 ± 2.41 (86.5–98.0)
Moderate OSAS (n = 22)	22.15 ± 4.23 (13.8–29.0)	13	9	48.09 ± 10.01 (26–70)	33.38 ± 4.31 (26.12–40.06)	91.34 ± 2.00 (87.0–94.5)
Severe OSAS (n = 67)	48.46 ± 16.06 (30.1–99.6)	45	22	49.16 ± 11.04 (26–78)	34.91 ± 5.81 (25.61–58.59)	88.43 ± 7.29 (40.0–94.5)
P	< 0.001			< 0.001	0.048	< 0.001

*OSAS* obstructive sleep apnea syndrome, *AHI* Apnea–Hypopnea Index, *BMI* body mass index

There was no statistically significant difference between the degree of obesity and NLR, PLR and SII values (p > 0.05) (Table 5).

**Table 5:** Comparison of NLR, PLR, SII with degree of obesity

Obesity degree	NLR Mean ± SD (min–max)	PLR Mean ± SD (min–max)	SII Mean ± SD (min–max)
Normal (n = 14)	1.98 ± 0.46 (1.46–3.03)	110.44 ± 26.51 (71.12–164.83)	553.32 ± 280.73 (240.40–1275.80)
Overweight (n = 44)	2.02 ± 1.14 (0.89–7.55)	110.01 ± 39.28 (54.00–232.85)	509.46 ± 265.76 (212.00–1585.70)
1. Degree Obesity(n = 56)	2.13 ± 1.50 (0.71–9.18)	106.69 ± 39.85 (41.34–238.10)	603.63 ± 499.96 (132.30–3003.20)
2. Degree Obesity (n = 39)	2.87 ± 3.92 (0.73–21.27)	116.70 ± 62.45 (1.46–420.62)	724.14 ± 987.94 (6.00–6427.10)
3. Degree Obesity (n = 18)	2.05 ± 0.87 (0.76–3.91)	119.18 ± 35.37 (62.78–184.17)	579.68 ± 246.43 (238.60–1138.20)
P	0.387	0.779	0.564

*SII* systemic immune-inflammation index, *NLR* neutrophil/lymphocyte ratio, *PLR* platelet/lymphocyte ratio

The mean NLR, PLR and SII values of patients with OSAS were statistically significantly higher than those without OSAS (p < 0.05). A positive correlation of 18% was found between the presence of OSAS and the SII value (p = 0.016) (Table 6).

**Table 6:** Comparison of OSAS presence by NLR, PLR and SII

OSAS	NLR Mean ± SD (min–max)	PLR Mean ± SD (min–max)	SII Mean ± SD (min–max)
Yes (n = 130)	2.40 ± 2.10 (0.73–21.27)	107.09 ± 39.16 (1.46–238.10)	614.01 ± 387.20 (6.00–3003.24)
No (n = 41)	1.59 ± 0.77 (0.71–4.41)	125.27 ± 56.50 (56.84–420.62)	454.94 ± 280.30 (132.34–1800.25)
P	0.017	0.022	0.016

*OSAS* obstructive sleep apnea syndrome, *SII* systemic immune-inflammation index, *NLR* neutrophil/lymphocyte ratio, *PLR* platelet/lymphocyte ratio

No statistically significant difference was found when comparing OSAS severity and NLR, PLR and SII values (p > 0.05) (Table 7).

**Table 7:** Comparison of OSAS Severity by NLR, PLR and SII

OSAS severity	NLR Mean ± SD (min–max)	PLR Mean ± SD (min–max)	SII Mean ± SD (min–max)
Simple Snoring (n = 41)	1.59 ± 0.77 (0.71–4.41)	125.27 ± 56.50 (56.84–420.62)	454.94 ± 280.30 (132.34–1800.25)
Mild OSAS (n = 41)	2.62 ± 3.16 (0.76–21.27)	108.99 ± 46.15 (1.46–232.85)	586.72 ± 341.32 (6.00–1765.43)
Moderate OSAS (n = 22)	2.16 ± 1.43 (0.73–7.55)	104.99 ± 44.62 (41.34–238.10)	566.24 ± 313.60 (182.01–1585.65)
Severe OSAS (n = 67)	2.34 ± 1.38 (1.11–9.18)	106.62 ± 32.70 (62.78–215.13)	646.40 ± 434.56 (244.90–3003.24)
P	0.085	0.148	0.075

*OSAS* obstructive sleep apnea syndrome, *SII* systemic immune-inflammation index, *NLR* neutrophil/lymphocyte ratio, *PLR* platelet/lymphocyte ratio

## Discussion

We evaluated the correlation between OSAS and SII as well as NLR and PLR parameters to evaluate the relationship between OSAS and systemic inflammation and whether there is a change in inflammation markers with OSAS severity. We found a significant relationship between the presence of OSAS diagnosis and SII, NLR and PLR. However, we observed that these parameters did not change with OSAS severity.

More than one mechanism is considered responsible for the pathophysiology of OSAS. The first of these is chronic systemic inflammation. It has been shown that Kappa B, which regulates inflammatory gene expression in chronic inflammation, is activated by hypoxia and reoxygenation, resulting in changes in the number of neutrophils and monocytes, especially an increase in neutrophil count. The decrease in lymphocyte count, which has been associated with uncontrolled inflammatory pathways, has been shown to cause activation of the hypothalamic–pituitary–adrenal axis, likely increased systemic cortisol production and changes in sleep habits [6–8].

Chronic intermittent hypoxia increases sympathetic activity. Increased cortisol levels as a result of increased sympathetic activity cause an increase in lymphocyte count [9, 10]. On the other hand, neutrophils increase, lymphocytes decrease, and systemic inflammation occurs with the effect of endogenous catecholamines and cortisol after physiological stress. This systemic inflammation starts with the release of various proinflammatory cytokines (e.g. TNF-a, VEGF, IL-1 and IL-6) from related blood cells. Previous animal model studies have also reported that apnea and hypoxia in OSAS trigger leukocyte function and cause systemic inflammation [9–13]. WBC (White Blood Cell), neutrophil, lymphocyte and platelet activation have been reported to be a good indicator of inflammation in OSAS patients. These cells play a specific role in the process and regulate each other's functions by interacting during inflammation and thrombosis [6].

The SII index was first used as a marker to evaluate the relationship between cancer and inflammation, and its relationship with hepatocellular carcinoma in the postoperative period has been demonstrated [14]. Later on, it was used to evaluate the relationship between SII and prognosis in solid tumours such as colorectal cancer [15, 16]. SII has been shown in various studies to be associated with the atherosclerotic process besides the inflammatory process. The relationship between SII and disease severity in acute ischemic stroke patients has been investigated, and it has been reported that SII is an independent risk factor in indicating stroke severity [17].

On the other hand, to evaluate the effectiveness of SII in predicting prognosis, SII has been examined in patients who had sinus vein thrombosis, and it has been reported to be a potential predictor of poor prognosis [18].

Kim et al. evaluated the relationship between sleep parameters and systemic inflammation in 1102 patients with OSAS and found a statistically significant relationship between severe OSAS and SII [19]. Similarly, in our study, we found a statistically significant relationship between SII and the presence of OSAS (p = 0.016). Additionally, we did not find a statistically significant difference between the groups when we evaluated SII according to OSAS severity. Contrary to our study, Topuz et al., in a study of 194 OSAS patients, reported that there was a significant relationship between SII and OSAS severity [20]. Topuz et al. also divided patients into 4 groups according to their AHI values and observed a statistically significant difference in age, gender, body mass index and average oxygen saturation between the groups [20]. Similarly, we observed a statistically significant difference between the groups in age, body mass index (BMI) and average oxygen saturation values in the patients we included and divided into groups by AHI values.

In a study that analyzed the relationship between sleep disorders and systemic immune inflammation index, 8505 people in the US population were included, and it was emphasized that SII had a stronger relationship with sleep disorders than PLR and NLR. The study reported that sleep problems, OSAS symptoms, and daytime sleepiness were positively correlated with SII in US adults [21]. Other mechanisms responsible for the pathophysiology of OSAS are acute and chronic hypoxia causing MPV, PDW and HCT changes and sympathetic overactivity. Episodic hypoxemia, repetitive stimulation and increased inspiratory load cause many pathophysiological processes. It has been reported that OSAS patients are exposed to too much sympathetic load during the wake period due to this cycle. This sympathetic load is thought to trigger platelet activation with catecholamine discharge, thus causing changes in haematological indices such as MPV, PDW and PLR, and hypertension, creating endothelial damage, which triggers cardiovascular diseases [6].

Various evidence has been shown to suggest that susceptibility to cardiovascular disease in OSAS patients may be associated with endothelial dysfunction, excessive oxidative stress, and atherosclerosis triggered by increased systemic inflammation and sympathetic stimulation, and the studies have shown that OSAS is an independent risk factor for cardiovascular comorbidity [22–25]. Song et al. evaluated 290 patients diagnosed with OSAS, and they emphasized that PLR is associated with the severity of OSAS and is a systemic biomarker for hypertension [26].

During the inflammatory process, platelets are activated by increased aggregation and adhesion in platelets, leading to an increase in platelet volume (MPV). Since platelets are acute phase reactants, increased platelet count has been associated with the inflammatory process in studies in which increased MPV was found in OSAS patients [6, 9]. PLR, the ratio of platelets to lymphocytes is one of the haematological parameters and another marker used to show systemic inflammation. Platelets contain the cyclooxygenase (COX) enzyme to synthesize pro-inflammatory prostanoids such as thromboxane A2 and are a source of inflammation. In a study evaluating the role of COX pathways in OSAS, it was emphasized that intermittent hypoxia in mice increased COX-1 expression and thromboxane synthesis, causing atherosclerosis [27]. Additionally, Atan et al. showed that the number of thrombocytes increased significantly in OSAS patients as AHI, REM AHI, and NREM AHI increased and minimum oxygen concentration decreased [28].

Increased PLR predicts atherosclerosis that may occur in high-risk OSAS patients and, therefore, can help clinicians take timely precautions against cardiovascular risk in these patients. In our study, we observed a statistically significant difference between OSAS and PLR (p = 0.022). When we looked at the correlation between OSAS severity and PLR, we did not observe a statistically significant difference according to severity.

NLR is another marker used to show systemic inflammation.

NLR has been shown to be associated with many chronic diseases, such as pulmonary diseases and malignancy, as an indicator of the inflammatory process [29]. Topuz et al. also found a moderate statistical correlation (p < 0.05) between OSAS and NLR. They also argued that NLR may help predict the progression and prognosis of OSAS, but it alone is not sufficient [20].

In a study including 171 patients with OSAS, a correlation was found between OSAS and NLR, and it was also emphasized that it was an independent risk factor for cardiovascular disease. [30] Similarly, we observed a statistically significant difference between OSAS and NLR (p = 0.017). When we looked at the correlation between OSAS severity and NLR, we did not observe a statistically significant difference according to severity.

This study has several limitations. This was a retrospective, single-centre study conducted in a small group with a relatively small sample size. Multicenter prospective randomized controlled studies with larger case series are needed to achieve more statistical power and confirm our findings.

## Conclusion

In conclusion, we tried to reveal evidence of hypoxia, sympathetic activity, chronic systematic inflammation and oxidative stress, which are thought to be responsible for the pathophysiology of OSAS. We observed that SII, NLR and PLR parameters, which are rapidly assessable systemic inflammation markers of this process, were independently associated in patients diagnosed with OSAS. There was no change in SII, NLR, and PLR parameters with OSAS severity. We believe that detecting the presence of inflammation through these easily accessible, measurable, and low-cost methods can be useful in predicting the risk of complications such as cardiovascular disease that may occur in OSAS patients and in taking protective measures for them.
